# Deletion of Non-histidine Domains of Histidine Kinase *CHK1* Diminishes the Infectivity of *Candida albicans* in an Oral Mucosal Model

**DOI:** 10.3389/fmicb.2022.855651

**Published:** 2022-04-21

**Authors:** Yahui Feng, Shaodong Bian, Zhiping Pang, Yiyang Wen, Richard Calderone, Dongmei Li, Dongmei Shi

**Affiliations:** ^1^College of Clinical Medicine, Jining Medical University, Jining, China; ^2^Laboratory of Medical Mycology, Jining No. 1 People’s Hospital, Jining, China; ^3^Department of Pathology, Jining No. 1 People’s Hospital, Jining, China; ^4^Department of Microbiology/Immunology, Georgetown University Medical Center, Washington, DC, United States; ^5^Department of Dermatology, Jining No.1 People’s Hospital, Jining, China

**Keywords:** histidine kinase, *Candida albicans*, two-component signal transduction, fungal mucosal model, infectivity

## Abstract

**Objectives:**

The histidine kinase (HK) *CHK1* and other protein kinases in *Candida albicans* are key players in the development of hyphae. This study is designed to determine the functional roles of the S_Tkc domain (protein kinase) and the GAF domain of *C. albicans CHK1* in hyphal formation and mucosal invasion.

**Methods:**

The domain mutants CHK25 (^Δ*S_Tkc*^*CHK1/*Δ*chk1*) and CHK26 (^Δ*S_Tkc*Δgaf^*CHK1/*Δ*chk1*) were first constructed by the *his1-URA3-his1* method and confirmed by sequencing and Southern blots. A mouse tongue infection model was used to evaluate the hyphal invasion and fungal loads in each domain mutant, full-gene deletion mutant CHK21 (*chk1*Δ*/chk1*Δ), re-constituted strain CHK23 (*chk1*Δ*/CHK1*), and wild type (WT) from day 1 to day 5. The degree of invasion and damage to the oral mucosa of mice in each strain-infected group was evaluated *in vivo* and compared with germ tube rate and hyphal formation *in vitro.*

**Result:**

When compared with severe mucosal damage and massive hyphal formation in WT- or CHK23-infected mouse tongues, the deletion of S_Tkc domain (CHK25) caused mild mucosal damage, and fungal invasion was eliminated as we observed in full-gene mutant CHK21. However, the deletion of S_Tkc and GAF (CHK26) partially restored the hyphal invasion and mucosal tissue damage that were exhibited in WT and CHK23. Regardless of the *in vivo* results, the decreased hyphal formation and germ tube *in vitro* were less apparent and quite similar between CHK25 and CHK26, especially at the late stage of the log phase where CHK26 was closer to WT and CHK23. However, growth defect and hyphal impairment of both domain mutants were similar to CHK21 in the early stages.

**Conclusion:**

Our data suggest that both protein kinase (S_Tkc) and GAF domains in *C. albicans CHK1* are required for hyphal invasiveness in mucosal tissue. The appropriate initiation of cell growth and hyphal formation at the lag phase is likely mediated by these two functional domains of *CHK1* to maintain *in vivo* infectivity of *C. albicans*.

## Introduction

*Candida* species are the most common inhabitants of mucosal tissues that found in the oral cavities of up to 75% of the healthy population (Ghannoum et al., 2010). *Candida* species can be an opportunistic pathogen causing mucosal candidiasis due to the overuse of antibiotics, glucocorticoids, or immunosuppressive agents. Mucosal candidiasis, notably the oral, esophageal, and vaginal infections, is predominantly caused by *Candida albicans* to a lesser extent by other *Candida* species. Despite superficial mucosal infections are mostly non-lethal, their high frequency is found to be up to 80% (Pfaller and Diekema, 2010). Furthermore, in patients with immunosuppression, *C. albicans* damages the mucosal barrier and disseminates other organs with high mortality (Pfaller and Diekema, 2010).

Mucosal barrier disruption, neutropenia of the host, and fungal pathogenic traits, such as virulence factors, all elevate the risk of systemic candidiasis (Mayer et al., 2013). A wide range of fitness attributes of *C. albicans*, which are shared among *Candida* species, are key promoters in assisting the fungal invasion process in the mucosal layer. Notably, among these traits are a rapid adaptation to environmental fluctuations in nutrient availability and robust stress response machineries, and the yeast-to-hyphal morphological transition (Wolanin et al., 2002). The morphological plasticity of *C. albicans* is strongly associated with more than 100 proteins, among which protein kinases, two-component signal transduction proteins, and mitogen-activated protein kinase (MAPK) are known to be essential for the formation of hyphae in *Candida* (Das et al., 2019).

A two-component signal transduction system has been adopted in fungi to help them deal with external stresses, and this system operates through the stress-induced phosphorylation cascade response. Structurally, the prototypical two-component system is composed of a histidine kinase (HK) protein and a response regulator (RR) protein in bacterial species. In fungi, both proteins can be hybridized together to form a hybrid protein, such as HK protein (Wolanin et al., 2002). In a phosphorylation relay system, auto-phosphorylated histidine upon the perception of an environmental cue transfers the phosphoryl group to a conserved aspartate residue (D) within a receiver domain of its cognate RR protein. The output of the RR protein phosphorylates the downstream protein substrates and eventually initiates the transcription of a specific gene set. Three HK proteins have been found in *C. albicans*, namely, Chk1p, Nik1p, and Sln1p. Unlike other two HK proteins are preserved in most *Candida* species, such as *Cryptococcus neoformans*, *Aspergillus fumigatus*, and non-pathogenic *Saccharomyces cerevisiae*, Chk1p is found only in *C. albicans* and a few fungal species (Li et al., 2010).

The major function of a hybrid HK is carried out by three domains, namely, the HisK (H), ATPase, and REC (cheY receiver) domains, which are shared in Chk1p, Nik1p, and Sln1p at their C-terminus as predicted by SMART domain architecture analysis (Schultz et al., 2000). Although early studies have indicated that *C. albicans* utilizes HK proteins to sense stresses, to replicate, to undergo morphological transitions *in vitro*, and to regulate adhesion and tissue invasion during the *in vivo* infection (Calera and Calderone, 1999a; Calera et al., 1999b; Li et al., 2002, 2004), the degree of impairment varied according to the gene deletion. Specifically, full-gene deletion of the *NIK1* and *SLN1* genes only partially reduced hyphal formation and virulence, while the deletion of the *CHK1* gene caused a complete loss of both virulence and hyphal formation *in vitro* and *in vivo* (Calera and Calderone, 1999a; Calera et al., 1999b; Yamada-Okabe et al., 1999). Apparently, Chk1p plays a more critical role in the regulation of virulence and morphological switching than do the other two HK proteins, and this asymmetry, in turn, indicates that these effects are not a direct result of the conserved domains they share (H-ATPase-REC).

The typical HK is a transmembrane receptor with an N-terminal extracellular sensing domain and a C-terminal cytosolic signaling domain, as shown in *C. albicans* Sln1p (Li et al., 2010). Unlike the typical transmembrane domain of Sln1p and the HAMP domain of Nik1p for putative sensory co-factor binding (Bibikov et al., 2000), the sensing domain of Chk1p has never been characterized. When compared with the 1,081 aa-sized Nik1p and the 1,373 aa-sized Sln1p, Chk1p is a large size of protein composed of 2,471 amino acids. In addition to the abovementioned three universal domains, Chk1p has one Ser/Thr kinase domain (S_Tkc, 435 aa–636 aa), one AAA-16 domain (ATPase, 689 aa–894 aa), and one GAF domain (cGMP-adenylyl cyclase-*Escherichia coli* FhlA, 1828–1975) on the N-terminus. Hybrid HK proteins with those three unique domains are found in *Schizosaccharomyces pombe* Mak2 and Mak3 for peroxide sensation (Catlett et al., 2003) and in *Penicillium marneffei* as well, a common fungal pathogen found in HIV-infected lung tissue (Wong and Wong, 2011). As a proposed sensor protein, the actual signals for Chk1p transduction remain unclear.

The HK proteins are broadly conserved and not found in humans, a fact which then affords us not just an excellent target but a new field of possible targets for antifungal discovery. This study possibly begins the long process of achieving a full understanding of the biological characteristics of fungal HKs and their mucosal invasion abilities. For this objective, we use a mucosal infection model to test the importance of S_Tkc, AAA-16 (ATPase), and GAF domains of pathogenesis. Since this ATPase domain is functionally linked to a protein kinase in many organisms, we will use the S_Tkc domain deletion symbol to represent both S_Tkc and AAA-16 deletion in CHK25 in this study. Our data demonstrate that a complete deletion of the S_Tkc and AAA-16 domains in CHK25 remarkably reduces morphological switching during a mucosal invasion *in vivo*. The additional GAF deletion in CHK26 partially restores the hyphal defects observed in CHK25 and, therefore, represents an intermediate impairment in tissue invasion. Thus, our data suggest that N-terminal domains of the CHK1 gene are as important as the C-terminal enzymatic domains for hyphal formation during infection and *in vitro* growth.

## Materials and Methods

### Gene Deletion Mutant Source and Domain Mutant Construction

The *CHK1* gene deletion mutant CHK21 (*chk1*Δ*/chk1*Δ) and reconstituted strain CHK23 (*CHK1/chk1*Δ) were previously constructed in Dr. Calderone’s group (Table 1) in the Department of Microbiology/Immunology, Georgetown University (Calera and Calderone, 1999c). Disruption of the *CHK1* gene domain was performed according to the URA-blaster protocol using the lithium acetate transformation method (Fonzi and Irwin, 1993; Calera and Calderone, 1999c), and the genotypes of each strain are shown in Table 1. Strain *C. albicans* SC5314 as wild type (WT) and together with CHK23 (*CHK1/chk1*Δ) was used as controls for all experiments.

**TABLE 1:** Histidine kinase mutants and domain mutants of *C. albicans* used in this study.

Strain	Relevant genotype	Source/references
SC5314 (WT)	Wild type strain, *URA3/URA3* *CHK1/CHK1*	Fonzi and Irwin, 1993
CHK21	*ura3*Δ*::*λ*imm434/ura3*Δ*::*λ*imm434 chk1*Δ*::hisG/chk1*Δ*::hisG-URA3-hisG*	Calera and Calderone, 1999c
CHK22	*ura3*Δ*::*λ*imm434/ura3*Δ*::*λ*imm434 chk1*Δ*::hisG/chk1*Δ*::hisG*	Calera and Calderone, 1999c
CHK23	*ura3*Δ*::*λ*imm434/ura3*Δ*::*λ*imm434 chk1*Δ*::hisG/CHK1*	Calera and Calderone, 1999c
CHK25	*ura3*Δ*-IRO1::*λ*imm434/ura3*Δ*::*λ *imm434 chk1*Δ*::hisG/CHK1-S_Tkc*Δ	This work
CHK26	*ura3*Δ*-IRO1::*λ*imm434/ura3*Δ*-IRO1::*λ*imm434 chk1*Δ*::hisG/CHK1-S_Tkc*Δ*-gaf*Δ	This work

**FIGURE 1:**
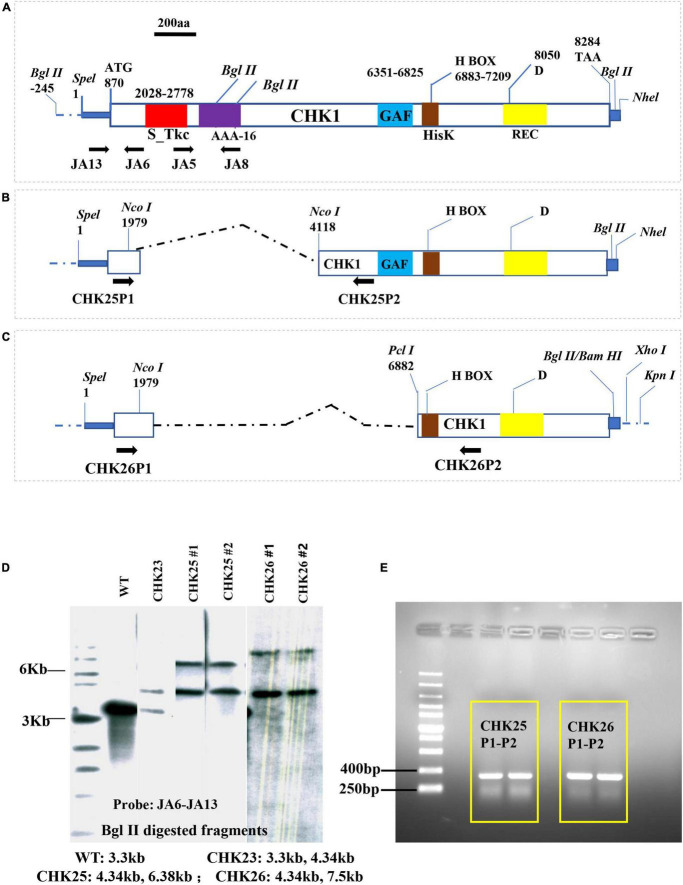
Plasmid construction for *CHK1* gene domain deletions. The entire OPF of the *CHK1* gene with domain architecture **(A)** is maintained at plasmid pChk1, which was constructed by inserting a *Spe*I-*Nhe*I fragment into PbsII sk^+^ *Spe*I cloning site. Two plasmids, namely, pChk2 **(B)** and pChk3 **(C)**, were derived from pChk1 to delete S_Tkc domain (red box), AAA-16 (purple box) in CHK25, and S_Tkc domain, AAA-16, and GAF domains (blue box) in CHK26, respectively. Numbers represent nucleotide positions of each function domain or important residue in CHK1. **(D)** Southern hybridization confirmation of two transformants of CHK25 (#1 and #2) and CHK26 (#1 and #2) was done with a probe generated from JA6 and JA13 primers, as shown in Table 1. The expected sizes of genomic *Bgl*II fragments for CHK25 (^Δ*S_Tkc*^*CHK1/*Δ*chk1*), CHK26 (^Δ*S_Tkc*Δgaf^*CHK1/*Δ*chk1*), CHK23 (*CHK1/chk1*Δ), and WT are shown. **(E)** PCR confirmation for CHK25 and CHK26 transformation using sets of CHK25P1-CHK25P2 and CHK26P1-CHK26P2 listed in Table 1. S_Tkc, serine/threonine kinase-like domain; AAA-16, ATPase; GAF, cGMP-adenyl cyclase-*E. coli* FhlA; HisK (brown box), histidine kinase domain; REC, response receiver domain; D, predicted phosphorylating aspartic acid.

Construction of CHK25 (^Δ*S_Tkc*^*CHK1/*Δ*chk1*) was accomplished by the transformation of plasmid pChk2 into CHK22 (*chk1*Δ/*chk1*Δ; *ura3*Δ*/ura3*Δ) (Table 1). Construction of pChk2 was accomplished by ligation of two DNA fragments excised from pChk1R and cloning into a *Spe*I/*Bam*HI-digested pBSIIsk^+^ vector. The template plasmid pChk1R containing entire 2,470 amino acids of CHK1 open reading frame (ORF) and 870 bp promoter on 5′ end and 24 bp after TAA code. The *Kpn*I/*Sal*I fragment of pMB7 containing *his1-URA3-his1* cassette (3.9 kb) was then inserted into *Kpn*I/Xhol-linearized pChk2 at 3′ flanking side of *CHK1*. As shown in Figure 1, two fragments of pChk1R were individually excised with *Spe*I/*Nco*I (promoter plus first 371 aa) and *Nco*I/*Bgl*II (after 1,083 aa) endonucleases and nicely fused together without frameshift, which resulted in the deletion of the Ser/Thr domain (at a position of 387 aa–634 aa). The recombination in CHK22 was allowed by ∼750 bp upstream Hid III site at the 5′ end and 91 bp at 3′ end that has been induced within the CHK1 gene deletion cassette in a previous study.

A similar strategy was used for the construction of CHK26 (^Δ*S_Tkc*Δgaf^*CHK1/*Δ*chk1*) with *Kpn*I/*Spe*I-linearized pChk3 plasmid transformation. The pChk3 was generated by a fusion of the same *Spe*I/*Nco*I DNA fragment (1/1,979) and *Pci*I/*Bgl*II fragment of pChk1R (6,882/8,307) with the *Spe*I/*Bam*HI sites of the pBSIIsk + vector. This pChk3 contains 1,115 bp *CHK1* promoter, the 371 amino acids upstream of s/t MAPK domain, and amino acids sequence after amino acid at 2,004 position, resulting in deletion of the Ser/Thr domain and GAF domain (6,603 aa–7,060 aa). The same *his1-URA3-his1* cassette (3.9 kb) was then subcloned into *Kpn*I/Xhol sites of the pChk3 at 3′ flanking side of *CHK1* and allowed to transform in CHK22.

### The Effect of Deletion of *CHK1 on* the Fungal Growth *in vitro*

The 5 strains (Table 1), namely, SC5314(WT), CHK21 (*chk1*Δ*/chk1*Δ), CHK23 (*CHK1/chk1*Δ), CHK25 (^Δ*S_Tkc*^*CHK1/*Δ*chk1*), and CHK26 (^Δ*S_Tkc*Δgaf^*CHK1/*Δ*chk1*), were used to evaluate the fungal growth *in vitro*. First, all the 5 strains were grown in yeast extract peptone dextrose medium (YPD) medium [1% (wt/vol) yeast extract, 2% (wt/vol) peptone, 2% (wt/vol) dextrose] for 200 rpm overnight at 30°C and then harvested. Then, cell suspension was diluted into a 20 ml YPD medium at an initiation concentration of 1 × 10^6^/ml and cultured at 30°C (Zhang et al., 2018). The fungal growth in each 1 ml aliquot was measured by a spectrophotometer (OD_600_) at 20, 40, 60, 80, 100, 120, 240, 480, and 960 min.

### The Formation Rate of Strain Germ Tube and Hypha *in vitro*

First, we used Spider medium containing 10% fetal bovine serum at 37°C for 3 days in order to restore the ability of hyphal formation (Bedell and Soll, 1979). Second, the abovementioned strains were suspended into 5 ml of YPD broth and incubated overnight at 30°C with shaking at 200 rpm. After washing three times with phosphate-buffered saline (PBS), the collected yeast cells (1 × 10^6^/ml) were inoculated into 2 ml of Spider-serum medium at 37°C with shaking at 200 rpm. Third, 5 μl cell suspension was collected at 20, 40, 60, 80, 100, 120, 240, 480, and 960 min, and then mixed with 5 μl of calcofluor white (CFW) fluorescent dye to observe under fluorescent microscopy; randomly 10 fields were chosen to calculate the percentage of germ tubes or hyphal cells (germ tubes number/total cell number × 100%). The positive germ tube is defined by three times or greater length of hyphal formation than yeast cells, and the experiment was repeated 3 times.

### Invasive Ability of *C. albicans CHK1* Domain Mutants in Mouse Oral Mucosal Infection Model

We use a murine model of oral mucosal candidiasis under immunocompromised and immunocompetent conditions to assess fungal invasiveness. We follow an earlier report (Samaranayake and Samaranayake, 2001) but with some modifications. For the immunocompromised model, 6-week-old female C57BL/6 mice were injected at days 2 and 1 with cyclophosphamide (100 mg/kg) intraperitoneally to simulate an immunodeficiency state. At day 2 after the last injection, the mice were intraperitoneally injected with 10% chloral hydrate at 10 ml/kg to induce a coma status for about 90 min. From a total of 150 mice, 25 mice were used for each testing group as follows: SC5314 (WT), CHK21 (*chk1*Δ*/chk1*Δ), CHK23 (*CHK1/chk1*Δ), CHK25 (^Δ*S_Tkc*^*CHK1/*Δ*chk1*), and CHK26 (^Δ*S_Tkc*Δgaf^*CHK1/*Δ*chk1*) infection group and PBS control group. Each mouse was infected on the tongue with a cotton swab bearing 5 × 10^7^clonal formation unit (CFU) fungal cells. Five mice from each testing group were sacrificed at days 1–5 post-infection (dpi), and infected tongues were removed for evaluation. After photography, each infected tongue was longitudinally divided into two parts. Half of the tongue was weighed and then homogenized in 1 ml of aseptic PBS, and an aspirate of 50 μl suspension was then inoculated on Sabouraud dextrose agar (SDA) medium containing chloramphenicol for 48 h at 30°C to evaluate fungal load in tongue tissues. The remaining portion of each tongue was preserved for histopathological examination as below.

### Histopathological Study

Mouse tongue tissues were fixed immediately with 10% neutral formaldehyde fixative. The fixed tissues were dehydrated and embedded in paraffin to make 0.4-μm-thick paraffin sections, which were stained with periodic acid-Schiff staining (PAS) (Samaranayake and Samaranayake, 2001). The fungal biofilm thickness formed by different *Candida* species was estimated by measuring the deepest invasive distance from the mucosal surface. There were 12 measurements in PAS staining slides from each mouse, and the average value was taken.

### Statistical Analysis

The SPSS version 23.0 software was used for statistical analysis. Differences between groups were tested and compared using the independent sample *t*-test and Tukey’s test in one-way analysis of variance (ANOVA). The value of *p* < 0.05 is considered statistically significant, and the symbol of “^***^” denotes for *p* < 0.001, “^**^” for *p* < 0.01, and “*” for *p* < 0.05 for all the graphs in this study.

## Results

### Confirmation of Chk1p Domain Mutants

The structure and domain architecture of the *CHK1* gene are shown in Figure 1, in which the cassettes pChk2 (Figure 1B) used for CHK25 (^Δ*S_Tkc*^*CHK1/*Δ*chk1*) construction and pChk3 (Figure 1C) for CHK26 (^Δ*S_Tkc*Δgaf^*CHK1/*Δ*chk1*) construction, and the upstream and downstream primers for Southern blot and PCR verification are all included. The genomic DNA of the mutant strain was extracted with a fungal genome extraction kit (Omega Fungal DNA kit). In the Southern blot analysis, a radiolabeling of an [ɑ-32P] dCTP probe of 915 bp obtained with primer set of JA13-JA6 (Table 2) confirms the presence of transformants of CHK25 and CHK26 with *Bgl*II-digested genomic DNA in the final product, as shown in Figure 1D. The primers for PCR confirmation for pChk2 and pChk3, and CHK25 and CHK26 are also listed in Table 2. The size of the amplified products of ∼300 bp verifies the correct construction of CHK25 and CHK26 (Figure 1E), and negative control for PCR confirmation of CHK25 and CHK26 was performed by primer set of JA8 and JA5.

**TABLE 2:** Primer list for the probe and PCR confirmations of domain mutant cassettes.

Names of primer sets	Forward primer (5′-3′)	Reverse primer (5′-3′)	Size
Probe/JA13-6	CAGTATCTCTCA CCTAACGTACAG	GTACCACTCAT TAAGAAAAGCCG	915 bp
CHK25/ P1-P2	TGGTCCAATG AGTCTCTCACA	TGGAGTCTG GTCAGAGGC	2.4 kb for WT 363 bp for CHK25
CHK26/ P1-P2	TGGTCCAATGA GTCTCTCACA	CGTTATCATTG CGGAGCTCTGAAT	5.2 kb for WT 361 bp for CHK26
NegControl/JA8-5	GAGTATGGAG TTTCCAAATCC	GGGGATTG TTTCCCC	632 bp for WT Negative for CHK25, CHK26

### Growth Defects of Two Domain Mutants (CHK25 and CHK26) at the Early Growth Stage Are Similar to Full-Gene Deletion Mutant CHK21

**FIGURE 2:**
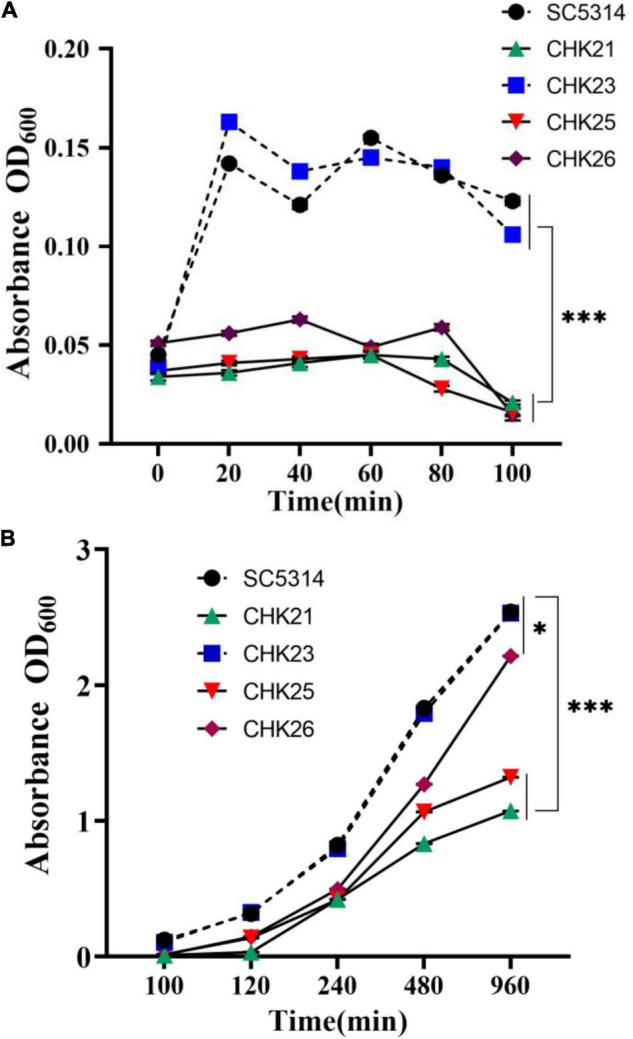
Growth curves of WT (SC5314) and every mutant strain in YPD broth were generated by OD_600_ absorbance at 20 min intervals. The initial concentration was 10^6^ cells/ml. Fungal cell proliferation from 0 to 100 min **(A)** and 100–960 min **(B)** show the lag phase and log phase growth, respectively. Each value is the average of three independent determinations with standard deviations of 4–15%. The domain mutants CHK25 (^Δ*S_Tkc*^*CHK1/*Δ*chk1*) and CHK26 (^Δ*S_Tkc*Δgaf^*CHK1/*Δ*chk1*) have the same growth deficits as full-gene mutant CHK21 (*chk1*Δ*/chk1*Δ) during the entire lag phase with significant differences when compared with WT (SC5314) or *CHK1*-reconstituted CHK23 (*CHK1/chk1*Δ). The *p*-values for 20, 40, 60, 80, and 100 min are all below 3.17 × 10^–11^. The growth defects of CHK21 and CHK25 continue over 100–960 min with the *p*-values below 3.53 × 10^–15^. However, the growth defect of CHK26 was partially restored during its log phase. At 960 min post-inoculation, the difference between CHK26 and WT or CHK23 is reduced (*p* = 0.021). *P*-values among groups have been determined where “^***^” denotes *p* < 0.001, and “*”denotes *p* < 0.05 for all graphs in this study.

The proliferation cycle of microbes including *C. albicans* can be divided into three phases, namely, the lag phase, the exponential phase, and the stationary phase. To understand the biological function of S_Tkc and GAF domains in Chk1p, the growth curves of CHK25 (^Δ*S_Tkc*^*CHK1/*Δ*chk1*) and CHK26 (^Δ*S_Tkc*Δgaf^*CHK1/*Δ*chk1*) were plotted against CHK21 (Δ*chk1/*Δ*chk1*), WT, and reconstituted strain CHK23 (*CHK1/*Δ*chk1*) based on OD_600_ absorbencies. Growth curve in nutrient-rich YPD broth at the lag phase (0–100 min) is shown in Figure 2A, and the exponential phase and the beginning of the stationary phase (100–960 min) are shown in Figure 2B. At the lag phase, we see a quick jump of OD_600_ absorbance from 0.03 to 0.15 within the first 20 min, and slight drops at 40 and 100 min are typically shown in WT and CHK23. The growth pattern shown in WT or CHK23 is disturbed in domain mutants CHK25 and CHK26 and null mutant CHK21 (Figure 2A). All three mutants retain OD_600_ absorbances around 0.05 throughout the lag phase, which is significantly different from SC5314 and CHK23 (*p* < 0.001). In the beginning of the exponential phase, the growth defects of the three mutants seem to be less apparent. As shown in Figure 2B, the growth patterns among all five strains are more similar to each other between 100 and 960 min than between 0 and 100 min. However, when compared with WT and CHK23, the growth defects still remain in CHK21 or CHK25 (*p* < 0.001) and are less affected in CHK26 (*p* < 0.05). These data suggest that both S_Tkc and GAF domains likely mediate the replication of *C. albicans*, especially during the initiation phase of growth.

### Germ Tube/Hyphal Formation Decreases in *CHK1* Gene Deletion and Domain Mutants

**FIGURE 3:**
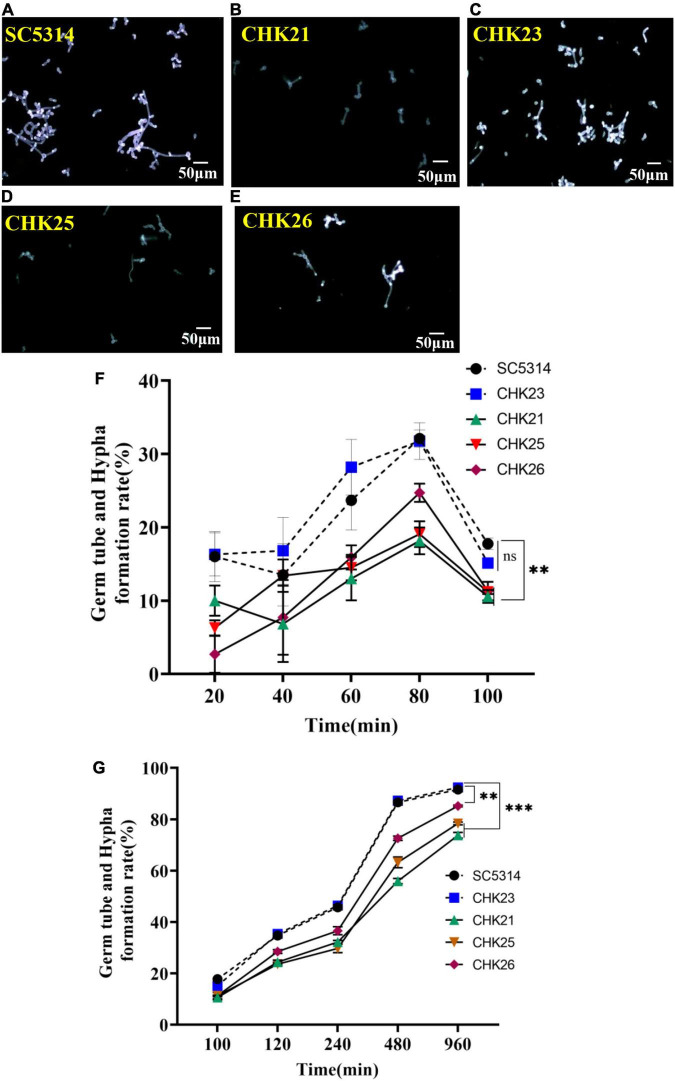
Germ tube formation in serum was highlighted with calcofluor white (CFW) staining at 80 min for each strain **(A–E)**. The short and poorly stained germ tubes in CHK25 (^Δ*S_Tkc*^*CHK1/*Δ*chk1*), CHK26 (^Δ*S_Tkc*Δgaf^*CHK1/*Δ*chk1*), and CHK21 (*chk1*Δ*/chk1*Δ) were contrasted to the strong fluorescence-stained germ tubes in SC5314 (WT) and CHK23 (*CHK1/chk1*Δ). The germ tube/hyphal formation rates were calculated over 20–100 min **(F)** and 100–960 min **(G)** in three independent experiments. There was no significant difference between SC5314 and CHK23 at any time point (*p >* 0.05). All three mutants (i.e., CHK21, CHK25, and CHK26) have significantly decreased hyphal formation rates versus WT or CHK23, with *p* < 0.001, except no difference detected between CHK25 and WT and CHK23 at 40 min (*p* = 0.270). During 100–960 min, the hyphal formation rates remain low in CHK 25 and CHK21, with *p* < 0.00001 at any time point versus WT, and decreased hyphal formation rates in CHK26 are less affected with *p* < 0.01. *P*-values among groups have been determined where “^***^” denotes *p* < 0.001, “^**^”denotes *p* < 0.01, and for all graphs in this study.

We followed the germ tube and hyphal formation *in vitro* for 960 min to monitor the impact of the *CHK1* gene on hyphal growth. As shown in Figures 3A–E, the germ tubes formed at 80 min are fewer in number, shorter, and only weakly staining by CFW in CHK21 (*chk1*Δ*/chk1*Δ), CHK25 (^Δ*S_Tkc*^*CHK1/*Δ*chk1*), and CHK26 (^Δ*S_Tkc*Δgaf^*CHK1/*Δ*chk1*) cultures. This weak staining on the cell wall of CHK25 (Figure 3D) is also observed in CHK21 (Figure 3B), and can be due to the cell wall defect. In our earlier study, the large and intermediate-sized mannoproteins are absent in null mutant CHK21 (Li et al., 2009). The germ tube formation rates within the first 100 min remain low for all strains with a slight peak at 80 min. The rate is ∼30% in WT and CHK23 (*CHK1/chk1*Δ) and immediately drops at 100 min to less than 20% for every strain in Figure 3F. There is no difference in germ tube formation rates between WT and CHK23 at any time (*p* > 0.05). However, pairwise comparisons show that the rates of germ tube formation within 960 min are universally decreased in the full-gene mutant CHK21 and in the domain mutants CHK25 and CHK26. When compared with WT and CHK23, the decrease in the hyphal formation rate in CHK21 and CHK25 is more obvious (*p <* 0.001) than CHK26 (*p <* 0.01) (Figure 3G).

### Gross Lesions and Fungal Loads Are Less Evident in Chk1p Mutant-Infected Mice

**FIGURE 4:**
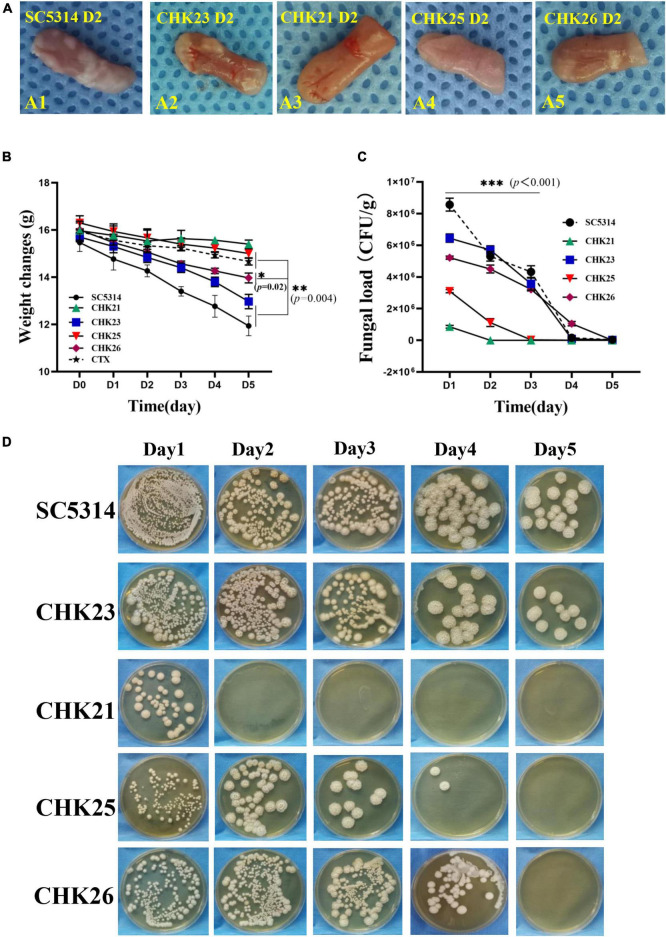
Gross appearance of tongue in immunodeficient mice at dpi 2 (D2) infected by SC5314, CHK21 (*chk1*Δ*/chk1*Δ), CHK23 (*CHK1/chk1*Δ), CHK25 (^Δ*S_Tkc*^*CHK1/*Δ*chk1*), and CHK26 (^Δ*S_Tkc*Δgaf^*CHK1/*Δ*chk1*) **(A)**. The white pseudomembrane and the areas of swelling are clearly shown in tongues infected by WT and CHK23 but are less apparent in tongues infected by null and domain mutants. **(B)** Body-weight changes in mice infected by 5 different *C. albicans* strains and control mice receiving cyclophosphamide (CTX). Values are means (*n* = 5) with standard errors for prior to infection dpi 0–5. Body weights are significantly reduced in WT and CHK23 at dpi 5 versus CTX control mice (*p* < 0.01) but not significantly reduced in CHK21, CHK25, and CHK26. **(C)** Fungal load (CFU/g) in tongue tissues of mice on different days post-infection with 5 *C. albicans* strains. Each data point represents mean ± SD of five values (*n* = 5) at each time point. **(D)** An aliquot of each homogenous tongue infected by different *C. albicans* strains in 50 μl was inoculated on SDA after culture at 30°C for 2 days, and CFU was calculated. *P*-values among groups have been determined where “^***^” denotes *p* < 0.001, “^**^”denotes *p* < 0.01, and “*”denotes *p* < 0.05 for all graphs in this study.

The tongues of five mice from each group were excised at 1–5 days post-infection (dpi 1–5) for evaluation (Samaranayake and Samaranayake, 2001). As shown for dpi 2 (D2 in Figure 4A), the infected areas were swollen or often covered with white pseudomembrane materials in the immunocompromised mice. When measuring the weight change during 5 days of infection, the average body weight of mice infected by WT and CHK23 significantly declines (Figure 4B) when compared with the cyclophosphamide (CTX)-treated mice group (*p* < 0.01). Weight loss is also seen in the CHK26 (^Δ*S_Tkc*Δgaf^*CHK1/*Δ*chk1*)-infected group (*p* < 0.05), but no weight loss is seen in mice infected by CHK21 (*chk1*Δ*/chk1*Δ) or CHK25 (^Δ*S_Tkc*^*CHK1/*Δ*chk1*) versus the CTX control group.

To better quantify fungal load from day 1 to day 5 post-infection and to estimate the extent of hyphal invasiveness in each infected tongue, ground-up tissues were cultured on SDA for fungal recovery (Figures 4C,D). For WT, the CFU value presents highest at day 1 in the tongue tissue and remains high at day 2, but significantly drops after day 3. At day 5, a small number of fungal cells still survive. The CFU values in CHK23 are similar to WT during the 5 days of infection (Figure 4C). For CHK21, the CFU is about eightfold less than WT at dpi 1, and no fungal cells are seen after dpi 2 (Figure 4C). The CFU value in CHK25 infection is only one-third of WT value at dpi 1, which quickly declines at dpi 2 and dpi 3, and is barely detectable through dpi 4–5. While the overall CFU values are significantly reduced in CHK 21- and CHK 25-infected mice from dpi 1 to 5 (*p* < 0.001), the CFU counts in CHK26-infected mice in dpi 2 and 3 are similar to the CHK23 group but less abundant at dpi 1 and diminished at dpi 5 (Figure 4D). Nevertheless, fungal persistence is shorter for the null mutant or domain mutants in mouse tongues than for WT and reconstituted strain CHK23. Since domain mutants are easily shed from mucosa, our data suggest that both S_Tkc and GAF domains in CHK1 are required for maintenance of *C. albicans* viability in mucosal tissue.

All our gross findings were not obvious in immunocompetent mice infected with any strain. Instead, slight swelling was only seen in WT- or CHK23-infected mouse tongues with a few fungal cells recovery in SDA counts (data not shown).

### Mucosal Barrier Destruction and Hyphal Invasiveness Are Eliminated in CHK25 Infection and Suppressed in CHK26 Infection

**FIGURE 5:**
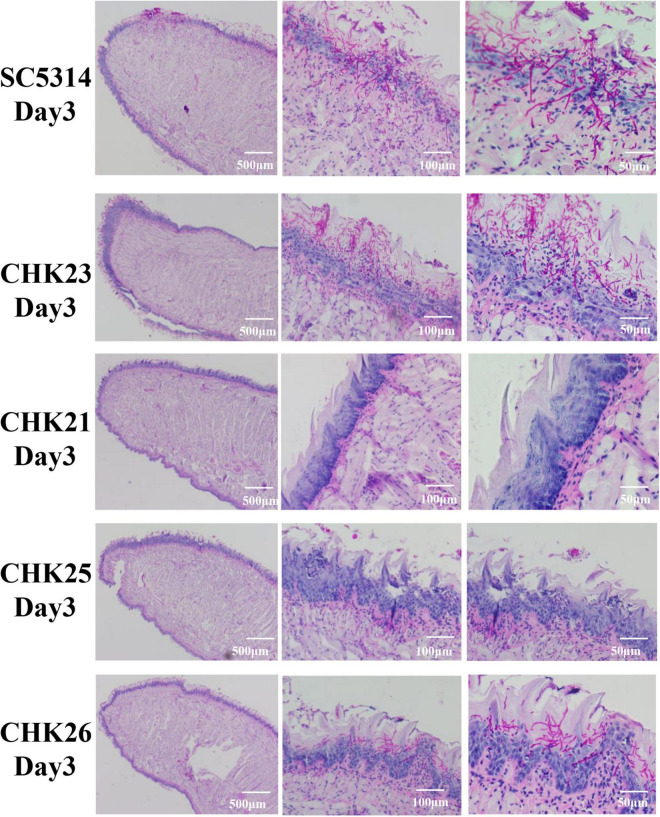
Histopathological presentations of infected tongues at day 3 post-infection for each fungal strain infection after PAS staining. Each scale bar in the figure denotes magnification and the magnification increases from left to right. Massive purple-red-stained hyphae penetrate the epithelial layer in SC5314- and CHK23-infected tongues, but are more restricted to the keratin layer in CHK26-infected tongues. Dominant yeast form of CHK25 presents on keratin layer with slight epithelial damage. Hyphal invasion/inflammation or mucosal damage is completely absent in CHK21-infected tongues.

The PAS staining was used to examine and compare the mucosal damage and the fungal invasion between control strain and each mutant-infected mouse group from dpi 1 to 5. Since CFU counts at dpi 3 seem to be a turning point for fungal clearance in mouse tissues (Figure 4C), we present pathological changes at dpi 3 in Figure 5 and dpi 1–5 listed in Supplementary Figure 1. Under low-power microscopy, PAS staining shows that the epithelial layer of mucosa in WT- and CHK23 (*CHK1/chk1*Δ)-infected tongues was more degraded than in any mutant-infected mouse group from dpi 1 to 5 (Figure 5 and Supplementary Figure 1), as indicated by the amorphous structure of the epithelia and the absence of the basal membrane in CHK23 and the missing keratin layer on the surface until dpi 5. The immune cell infiltrate is more often found in WT- and CHK23-infected epithelium (Supplementary Figure 1). In contrast, except for a slightly greater swelling in CHK25 (^Δ*S_Tkc*^*CHK1/*Δ*chk1*)-infected tongues than in CHK21 (*chk1*Δ*/chk1*Δ)-infected tongues, the mucosal barrier structures, such as keratin layer, remain intact in both CHK21-infected tongue, and better condition remains in CHK25-infected tongues (Figure 5). As with infections observed in CHK26 (^Δ*S_TkcΔgaf*^*CHK1/*Δ*chk1*), intermediate mucosal damages are found from dpi 1 to 5, and microabscess is more commonly in CHK26 than CHK25 in Supplementary Figure 1.

Under PAS staining, in agreement with the significant amount of swelling and pseudomembrane materials shown in WT- and CHK23-infected mice (Figure 4A), hyphal invasion *in vivo* is more severe on the tongues infected by WT and CHK23 than any time points by other three mutants (Figure 5 and Supplementary Figure 1). Massive hyphae penetrate through the epithelial, connective tissue, or even muscular layers in WT- or CHK23-infected mouse tongues at dpi 1 (Supplementary Figure 1). In contrast, no hyphal invasion is seen in CHK21- or CHK25-infected tongues, and the basement membranes are intact (Supplementary Figure 1). Most fungal cells of CHK21 and CHK25 are restricted to the keratin layer in yeast form at dpi 1. The hyphal form is detected at dpi 2 for CHK25-infected tongues, but not at any time point for CHK21-infected tongues. Apparently, the additional deletion of GAF domain in CHK26 leads to the partial recovery of hyphal formation function that shows in CHK26-infected tongues. We find a mixture of yeast and hyphae in CHK26-infected tongues at dpi 1 and then hyphal form of CHK26 dominates from dpi 2 to 4. At dpi 5, a few yeasts of CHK26 are restricted to the mucus layer in CHK26-infected tongues (Supplementary Figure 1). When compared with the massively long hyphae shown in WT- or CHK23-infected tongues, hyphal structures seem to be shorter and mixed with more yeast cells in CHK25 and CHK26. Furthermore, the basement membrane zone of the tongue tissue in the CHK26-infected area did not appear to be interrupted from dpi 1 to 5 (Figure 5 and Supplementary Figure 1).

**FIGURE 6:**
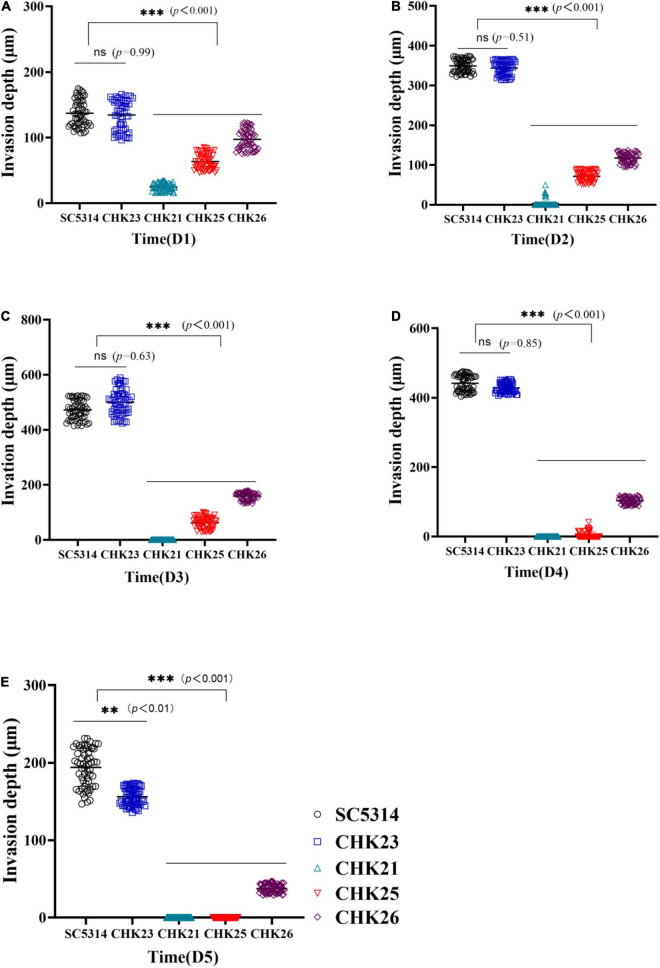
Hyphal invasion depth in PAS-stained infected tongue was determined by measuring the distance from the mucus layer interface or (if the mucus layer is no longer present) top surface to the greatest depth of fungal penetration in the epithelial and connective tissues. The means of 60 measurements obtained from five mice at dpi 1 **(A)**, dpi 2 **(B)**, dpi 3 **(C)**, dpi 4 **(D)**, and dpi 5 **(E)** are shown for statistical analysis; the black circles represent WT-infected tongues and bright blue rectangles represent CHK23-infected tongues; blue triangles and red triangles represent CHK21 and CHK25, respectively, and purple diamonds represent CHK26-infected tongues. *P*-values among groups have been determined where “^***^” denotes *p* < 0.001, “^**^”denotes *p* < 0.01, for all graphs in this study.

The extent of fungal invasiveness in WT, CHK23, and CHK26 was then quantified by measurement of invasive depth in PAS staining slides. Twelve sites were chosen in each PAS staining slide at random for the measurement of distance from the mucus layer interface or (if the mucus layer is no longer present) top surface to the greatest depth of fungal penetration in the epithelial and connective tissues, and averaged results are shown in Figures 6A–E for dpi 1 to 5. As shown in Figure 6, the invasive depths during dpi 1 to 5 are similar between WT- and CHK23-infected tongues. In agreement with the lower CFU count, the invasive ability was most impaired for the null mutant CHK21, and then for the CHK25 and CHK26 mutants versus WT- or CHK23-infected tongues (*p* < 0.001).

## Discussion

Invasive candidiasis is still responsible for most systemic fungal infections. The development of strategies to quickly and efficiently fight against this disease requires a better understanding of pathogenic traits and their impact on host immunity. HKs are the key signal transducer outside the animal kingdom (Wolanin et al., 2002). As a unique protein found in a few fungal species, *C. albicans*’ Chkp1 has been confirmed to be significant to many biological processes and pathogenic traits, including the regulation of cell wall glucan and mannan biosynthesis (Li et al., 2009), controlling population sensing (quorum sensing) (Kruppa et al., 2004), and mediating osmotic and oxidant stress responses (Li et al., 2004). The deletion of CHK1 reduces virulence in a murine disseminated model (Calera et al., 1999b) and in a mucosal model (Zhou et al., 2021), and has been explained as an effect of decreased expression of fungal virulence factors, including ALS2, SAP6, and YWP1 (Zhou et al., 2021).

S_Tkc, ATPase, and GAF are located at the N-terminus of Chk1p according to Pfam domain database analysis. As we mentioned above, studies from us and others have suggested that Chk1p plays a more critical role in *in vitro* hyphal formation and pathogenesis because full CHK1 gene deletion causes an avirulence phenotype. Whether these N-terminal domains are relevant to some putative sensory function of HKs has not been studied. In a previous study, Yamada-Okabe et al. (1999) compared the virulence changes in the three HK deletion mutants of *C. albicans*. They observed that the avirulence and absence of hyphae were only shown in Δ*chk1* mutant but not in two other HK mutants. In the deletion cassettes constructed for the three HK mutants, the C-terminal HisK-ATPase-REC was deleted in all three constructions. The resulting CHK1 gene deletion cassette included GAF domain but no N-terminal S_Tkc or AAA ATPase domains. Apparently, the residual S_Tkc and ATPase at the N-terminus could not complete the mucosal invasion alone, which must depend on enzymatic HisK function at the C-terminus of Chk1p. The second important discovery in the study of Yamada-Okabe et al. is that the HisK domain of Chk1p acts differently from the other two HKs in their catalytic activity, i.e., autophosphorylation. In the presence of the REC domain, when HisK domains in Sln1p and Nik1p were autophosphorylated, autophosphorylation failed completely in recombinant Chk1p in the presence of the same conserved HisK, ATPase, and REC domains. Apparently, the signal inducer, response pattern, and regulation of Chk1p enzyme activities are different from the other two HK proteins in *C. albicans*.

In this study, the lack of the S_Tkc and ATPase domains in CHK25 produces a similar avirulence phenotype to the full-gene deletion mutant CHK21 on mucosa, indicating the importance of this protein kinase domain to supplement those fully pathogenic traits of Chk1p. However, the additional removal of the GAF domain in CHK26 somehow partially restores hyphal formation both *in vivo* and *in vitro*. It seems that the function of S_Tkc and AAA ATPase is negatively affected by GAF regulation. Indeed, this partial attenuation of virulence and hyphal formation in CHK26 (residual of HisK-ATPase-REC) is similar to those of Δ*sln1* and Δ*nik1* (deletion of HisK-ATPase-REC) previously observed by Yamada-Okabe (Yamada-Okabe et al., 1999). If it can be inferred that the C-terminal of HK (HisK-ATPase-REC) accounts for the half-strength hyphal formation and virulence, the full-strength loss of virulence and hyphal formation in Δ*chk1* mutant constructed by Yamada-Okabe et al. (GAF and HisK-ATPase-REC) also highlights the importance of GAF domain in terms of maintaining the full function of Chk1p, including hyphal invasiveness in the host.

Fungal growth defects *in vitro* are less pronounced than *in vivo* for CHK25 (^Δ*S_Tkc*^*CHK1/*Δ*chk1*), CHK26 (^Δ*S_TkcΔgaf*^*CHK1/*Δ*chk1*), and CHK21 (*chk1*Δ*/chk1*Δ). However, distinct decreases, especially in the lag phase growth, were seen in the three mutant-infected mice with *p* < 0.001 versus WT or reconstituted strain CHK23 within 100 min post-inoculation. After 100 min, the slow growth in CHK26 becomes less apparent (*p* < 0.05), while CHK25 and CHK21 retain growth defects with *p* < 0.001. Taken together with the *in vivo* data, we speculate that at least S_Tkc and AAA ATPase participate in the initiation of fungal growth and hyphal formation, and this initiation is critical for a fast transition from lag to log phases. These *in vitro* growth data could be used to explain the infectivity loss of CHK25 *in vivo*. It is worth noting that during the lag phase, both WT and CHK23 show two small proliferation peaks at 20 and 60 min, and neither CHK21 nor CHK25 show similar bimodal proliferation peaks. Two smaller peaks occur in CHK26 but with 20 min delays. As for hyphal formation, the slight drop at 40 min and peak at 80 min in CHK23 are also shifted in CHK25 and CHK26. It seems that a lower hyphal formation rate at 80 min in CHK25 was the consequence of an unsuccessful attempt to limit hyphal formation at 40 min due to S_Tkc and AAA ATPase deletion. It is reasonable to contemplate that the growth regulation during the transition from the lag stage to the early log phase during the first 2 h of infection is the critical window for successfully establishing the infection in the host. The time line of *C. albicans* interacting with macrophages is well described by many studies. Typically, virtually engulfed *C. albicans* cells remain in the yeast form for 30 min post-infection, transitioning to hyphae inside phagolysosomes by 2 h (Westman et al., 2020). Hyphae continuingly grow in phagolysosomes and then disrupt the macrophage structures around 6 h post-infection (Jiménez-López and Lórenz, 2013). When we follow the procedures of Jiménez-López and Lorenz, we find that after 6 h, few point remains to the formation of hyphae: the battle is all but over. Thus, the low quantity of fungal cell population in the 80–100 min range in CHK21, CHK25, and CHK26 allows sufficient time for the host immune system to organize a proper immune counter attack and kill all invading fungal cells.

The mechanisms of S_Tkc and GAF in the regulation of HK enzymatic activity are beyond the scope of this study. They could be the signaling connectors among hybrid HKs as others have suggested (Yamada-Okabe et al., 1999), or between the phosphorelay pathway and other signaling pathways. Studies on HKs in plants and other organisms have suggested that HK signal transduction likely operates as a part of the transduction network. For example, it has been known that HKs control the activity of MAPK (Posas et al., 1996), HK signaling pathways in conjunction with G-protein, and even control intracellular cAMP levels through a downstream RR (Thomason et al., 1999).

The role of cyclic nucleotides has been well known for their regulatory role in fungal hyphal formation (Chow et al., 2021) and in other distinct biological functions in unicellular and filamentous bacteria (Schumacher et al., 2021). In other organisms, the GAF domain is commonly found in cGMP-specific phosphodiesterases, adenylyl cyclases, guanylyl cyclases, and phytochromes (McAllister-Lucas et al., 1993; Heikaus et al., 2009). GAF domains regulate gene transcription for light detection in bacteria and ethylene detection in plants. They also bind cGMP in phosphodiesterases, such as Pde5 (McAllister-Lucas et al., 1993). Furthermore, the GAF domain is found in formate hydrogenlyase transcriptional activator (FhlA) and NifA (transcriptional activator required for activation of most Nif operons for nitrogen fixation) (Santero et al., 1992). Structurally, two ATPase domains align with S_Tkc and HisK in Chk1p to likely separate the phosphorylation of downstream substrate proteins and autophosphorylation of histidine. The BLAST of the S_Tkc domain of Chk1p found it to appear in many proteins as well, if not in HK, and this domain sometimes links to ATPase, and is found in diguanylate cyclase and guanylate cyclase. It sometimes occurs in proteins containing diguanylate cyclase, PAS/PAC domain, and/or GAF sensors (Pandit et al., 2009). Whether the S_Tkc and GAF domains of Chk1p facilitate cyclic nucleotide signal-mediated morphological switching in *C. albicans* must await confirmation. Further studies will focus on the possible complex interactions between cGMP and cAMP signaling in balancing cell growth and hyphal formation in this pathogenic fungus.

## Data Availability Statement

The raw data supporting the conclusions of this article will be made available by the authors, without undue reservation.

## Ethics Statement

The animal study was reviewed and approved by Jining Medical University Animal Ethics Committee.

## Author Contributions

DL, RC, and DS contributed to the conception of the study. DL, YF, and SB performed the experiment. DL, YF, ZP, and YW performed the data analyses. DL, YF, RC, and DS wrote the manuscript. All authors contributed to the article and approved the submitted version.

## Conflict of Interest

The authors declare that the research was conducted in the absence of any commercial or financial relationships that could be construed as a potential conflict of interest.

## Publisher’s Note

All claims expressed in this article are solely those of the authors and do not necessarily represent those of their affiliated organizations, or those of the publisher, the editors and the reviewers. Any product that may be evaluated in this article, or claim that may be made by its manufacturer, is not guaranteed or endorsed by the publisher.

## Supplementary Material

Supplementary Figure 1 PAS staining of infected tongue tissues of mice at dpi 1 (D1), dpi 2 (D2), dpi 4 (D4), and dpi 5 (D5) post-infection with *C. albicans* SC5314 (WT), CHK23 (*CHK1/chk1*Δ), CHK21 (*chk1*Δ*/chk1*Δ), CHK25 (^Δ*S_Tkc*^*CHK1/*Δ*chk1*), and CHK26 (^Δ*S_TkcΔgaf*^*CHK1/*Δ*chk1*). The fungal invasion and mucosal damage are severe in CHK23- and WT-infected tongues until day 5. In contrast to ruptured mucosa and hyphal dominance in CHK23- and WT-infected tissues, shortened hyphae or a mixture with yeast-like cells are more commonly found in null mutant- and domain mutant-infected tongues.
