# Supplementary figures and images for: Deletion of Non-histidine Domains of Histidine Kinase CHK1 Diminishes the Infectivity of Candida albicans in an Oral Mucosal Model
Source: Front Microbiol. 2022 Apr 21;13:855651. doi: 10.3389/fmicb.2022.855651 (PMC9069115; doi:10.3389/fmicb.2022.855651)

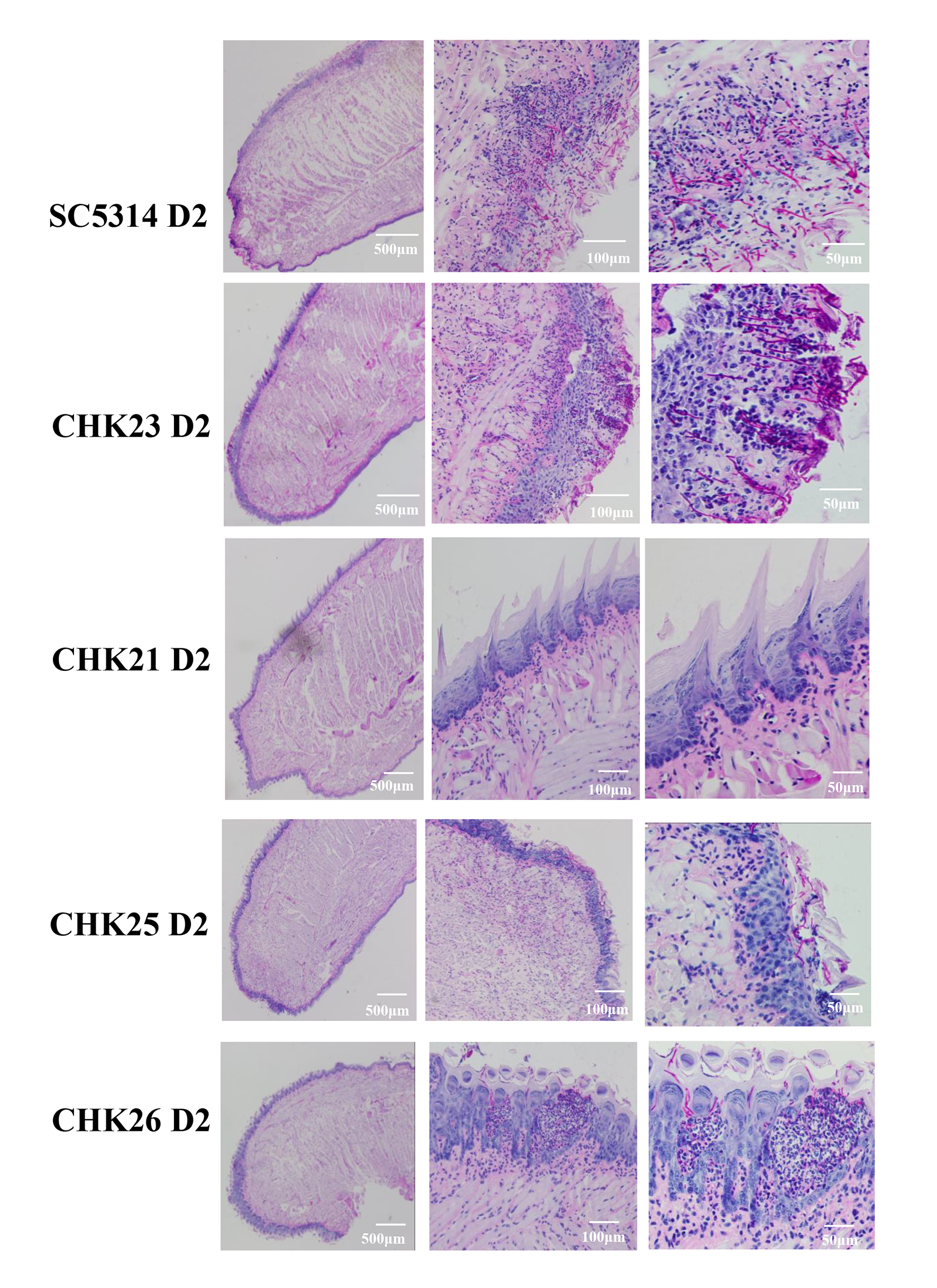

Supplement: Supplementary file 1 [file Image_2.TIF]

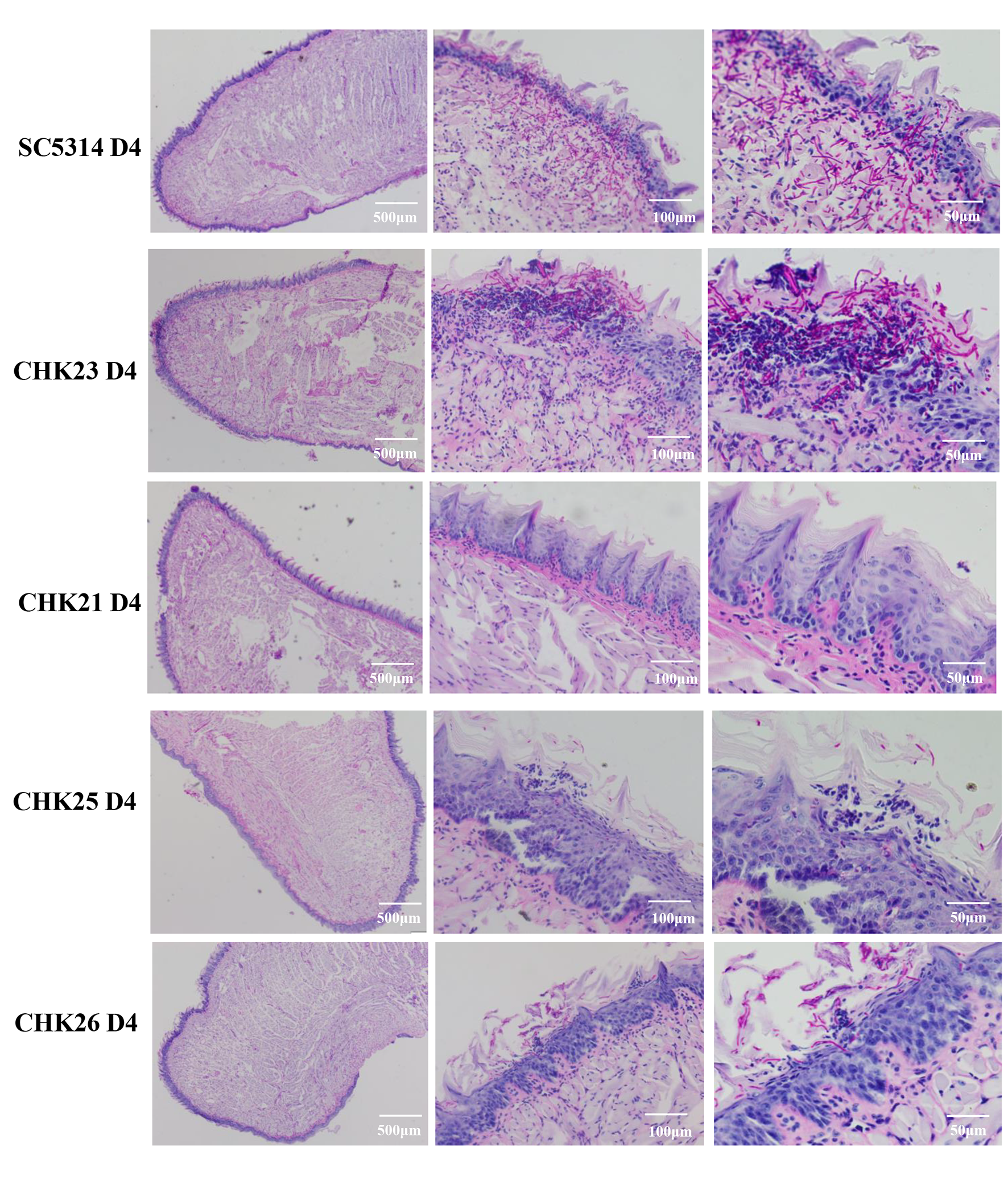

Supplement: Supplementary file 2 [file Image_3.TIF]

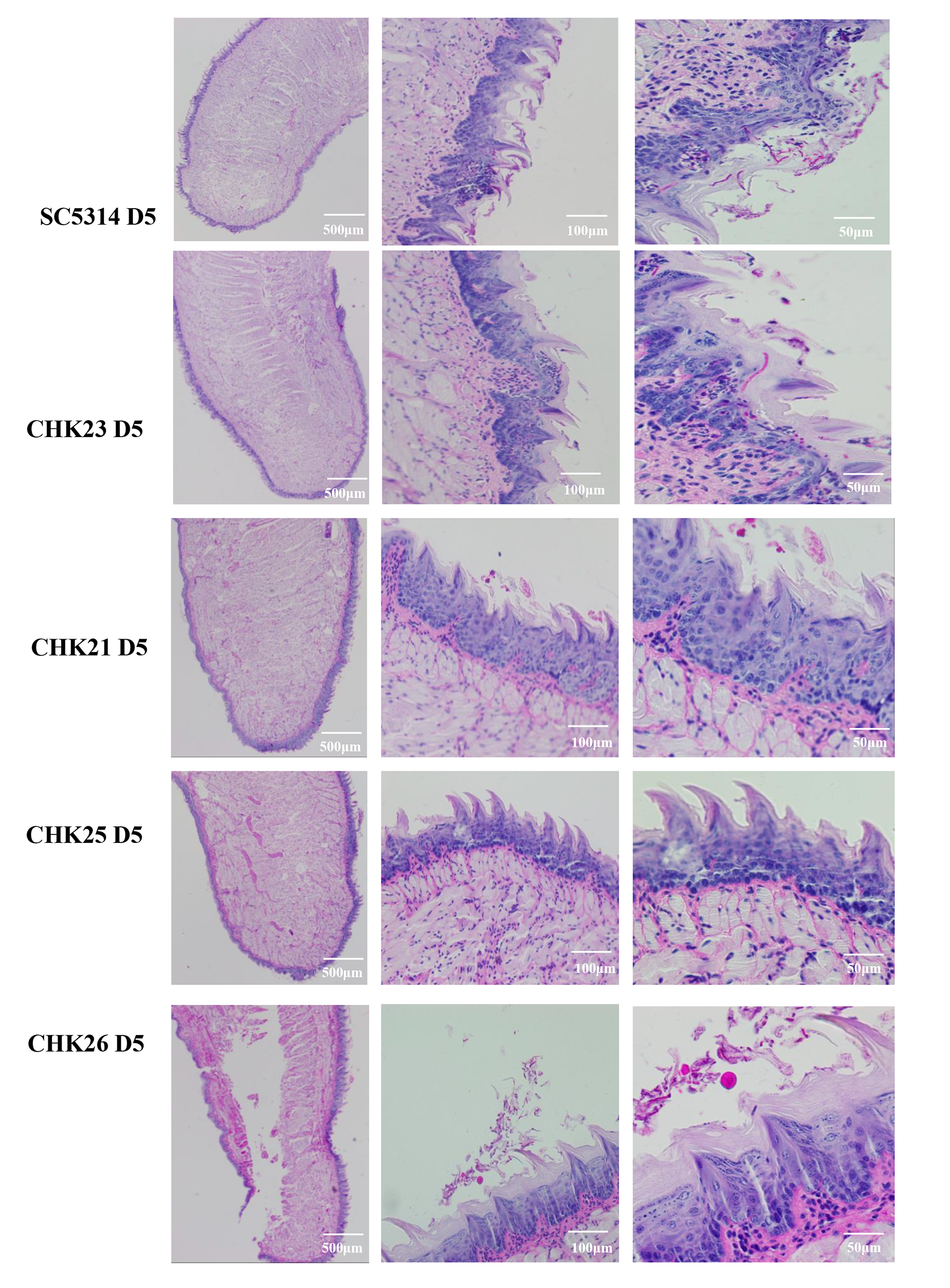

Supplement: Supplementary file 3 [file Image_4.TIF]

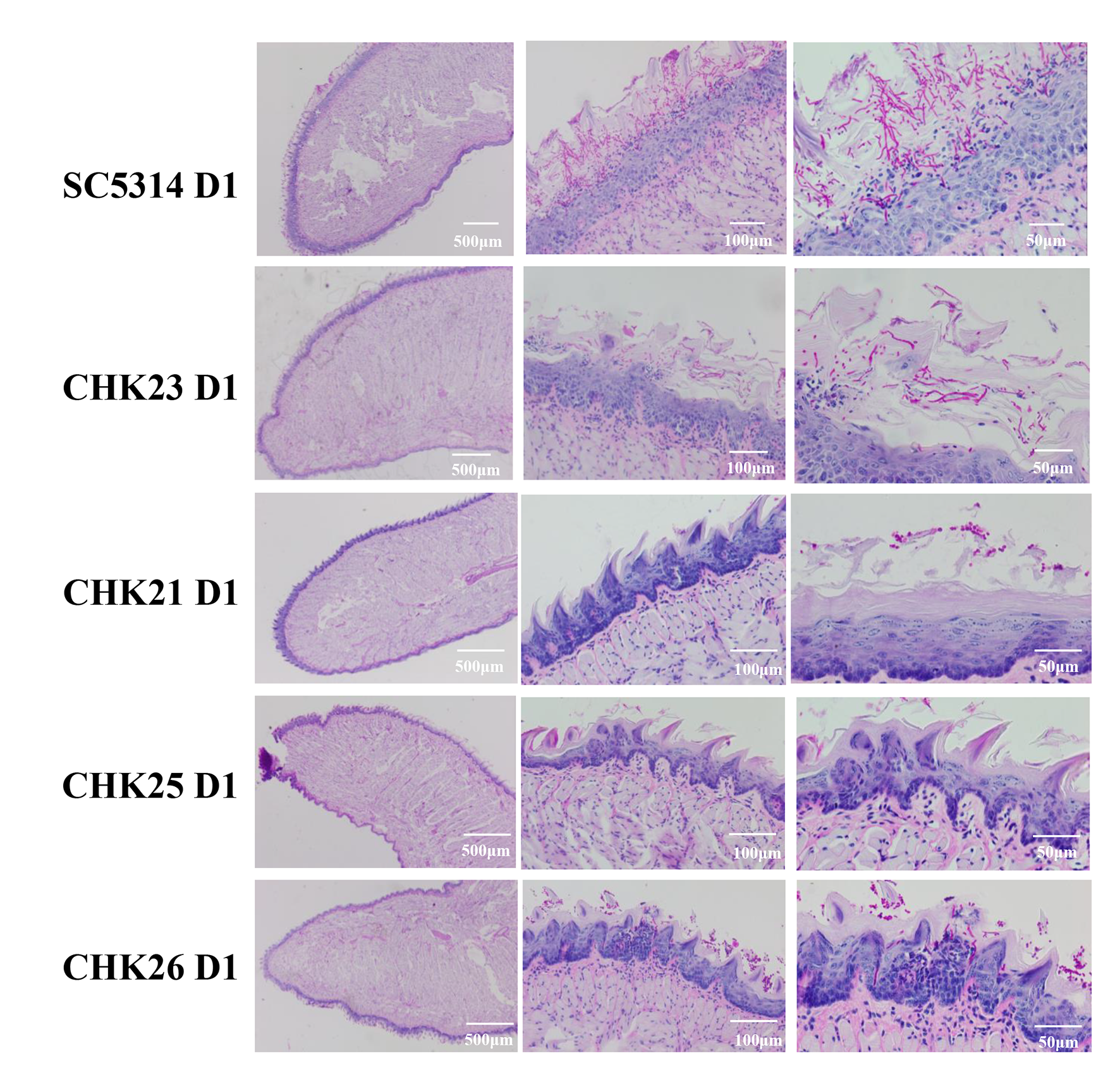

Supplement: Supplementary Figure 1 — PAS staining of infected tongue tissues of mice at dpi 1 (D1), dpi 2 (D2), dpi 4 (D4), and dpi 5 (D5) post-infection with C. albicans SC5314 (WT), CHK23 (CHK1/chk1Δ), CHK21 (chk1Δ/chk1Δ), CHK25 (ΔS_TkcCHK1/Δchk1), and CHK26 (ΔS_TkcΔgafCHK1/Δchk1). The fungal invasion and mucosal damage are severe in CHK23- and WT-infected tongues until day 5. In contrast to ruptured mucosa and hyphal dominance in CHK23- and WT-infected tissues, shortened hyphae or a mixture with yeast-like cells are more commonly found in null mutant- and domain mutant-infected tongues. [file Image_1.TIF]
